# LIGO Core-Collapse Supernova Detection Using Convolutional Neural Networks

**DOI:** 10.3390/s26061749

**Published:** 2026-03-10

**Authors:** Zhicheng Pan, El Mehdi Zahraoui, Patricio Maturana-Russel, Guillermo Cabrera-Guerrero

**Affiliations:** 1 Department of Electrical and Electronic Engineering, Auckland University of Technology, Auckland 1010, New Zealand; steven.pan@autuni.ac.nz; 2 Department of Mathematical Sciences, Auckland University of Technology, Auckland 1010, New Zealand; elmehdi.zahraoui@autuni.ac.nz (E.M.Z.); p.maturana.russel@aut.ac.nz (P.M.-R.); 3 Department of Statistics, University of Auckland, Auckland 1010, New Zealand; 4 Escuela de Ingeniería Informática, Pontificia Universidad Católica de Valparaíso, Valparaíso 2340025, Chile

**Keywords:** core-collapse supernovae, CNN, Q-transform, aLIGO

## Abstract

Core-collapse supernovae (CCSNe) remain a critical focus in the search for gravitational waves in modern astronomy. Their detection and subsequent analysis will enhance our understanding of the explosion mechanisms in massive stars. This paper investigates the use of convolutional neural networks (CNN) to enhance the detection of gravitational waves originating from CCSNe. We employ two time–frequency analysis techniques to generate spectrograms (training data): short-time Fourier transform (STFT) and Q-transform (QT). Two CNNs were trained independently on sets of spectrogram images of simulated CCSNe signals and advanced LIGO noise. The CNNs detect CCSNe signals based on their time–frequency representation. Both CNNs achieve a near 100% true positive rate for CCSNe GW events with a signal-to-noise ratio greater than 0.5 in our test set. Nevertheless, the CNN trained on the STFT spectrograms outperforms the one based on the Q-transform for SNRs below 0.5.

## 1. Introduction

Since Sir Isaac Newton, the nature of gravity has become one of the main subjects in physics. Newton initially described gravity as a force of attraction between masses in the 17th century. Newton’s law of universal gravitation revolutionised our understanding of celestial mechanics, explaining the motion of the moon and planets. However, the Newtonian framework had limitations, especially in explaining phenomena at cosmic scales. In the 20th century, Albert Einstein shifted our understanding of the nature of gravity by formulating the General Theory of Relativity [1]. This theory redefined gravity not as a force but rather as a mass curving spacetime, with spacetime dictating the movement of the mass. General relativity predicted phenomena that Newtonian physics could not account for, such as the bending of light by gravity, and described astrophysical dynamics precisely, such as the precession of planetary orbits. Einstein’s theory also led to the prediction of gravitational waves (GWs) that required a century of technological revolutions to validate empirically. GWs are ripples in spacetime that emerge from the universe’s most violent and energetic processes, such as the mergers of black holes and neutron stars [2]. GWs carry information about their origins and the nature of gravity, providing a novel method for observing and understanding the universe.

**Figure 1:**
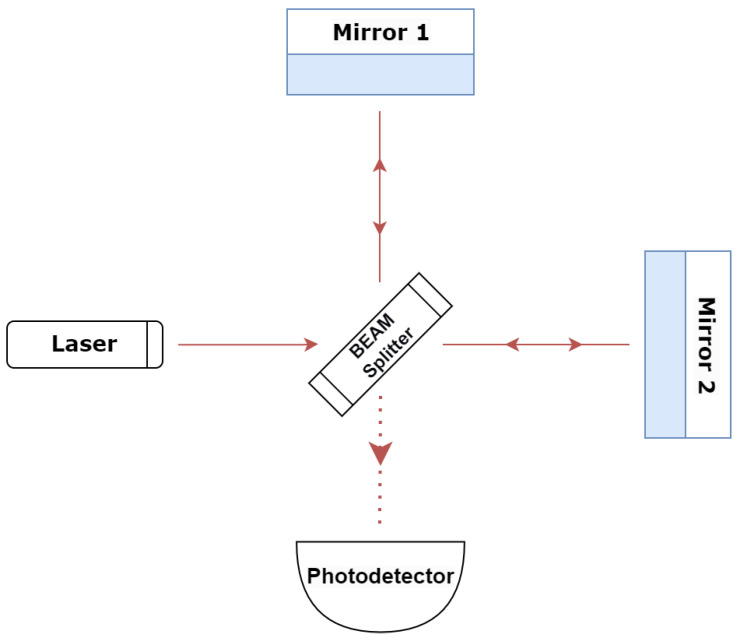
A simplified LIGO diagram with two perpendicular interferometry arms.

The first GW detection, resulting from the merger of a pair of black holes with masses of approximately 36 and 29 solar masses, was announced on 11 February 2016 [3]. The event was observed on 14 September 2015 by the Advanced Laser Interferometer Gravitational-Wave Observatory (aLIGO)’s Livingston and Hanford observatories, validating a crucial prediction of Einstein’s general relativity theory and offering the inaugural direct proof of black hole mergers. LIGO detectors are GW interferometers based on Michelson’s interferometer experiment. The LIGO interferometer is an optical instrument that splits a laser beam into two perpendicular arms and uses mirrors to reflect the laser beams to the photodetectors (see Figure 1). Interference between two laser beams will occur if the length of one of the arms or both changes, which will induce a phase shift in the laser beams. This phase shift is used to detect changes in distance. LIGO has approximately 4 km long arms with a GW characteristic strain sensitivity up to about $10^{-23}$. The ground-based interferometer can detect GWs in the 200 Hz–10 kHz frequency range [4]. Detection of GWs in a lower frequency range will be possible through LISA, a space-based interferometer built to observe in the µHz–Hz frequency range [5]. LISA is scheduled to launch by 2034. For even lower frequencies, the pulsar timing array (PTA) extends the sensitivity to the nHz–µHz range, helping to understand the early dynamics of astrophysical events [6].

aLIGO has been successful over the last decade in detecting and probing the GWs of multiple astrophysical phenomena [7,8]. However, despite being one of the main targets of these interferometers, the GW signals from Core-Collapse Supernovae (CCSNe) have yet to be detected. A core-collapse supernova is a powerful and catastrophic stellar explosion that occurs at the end of a massive star’s life cycle, when the core of the star, typically composed of iron, collapses under its own gravity, emitting high-luminosity light. Its luminosity can be brighter than the moon’s brightness and can last for a few weeks before fading away. This phenomenon is considered among the most powerful explosions in the universe, releasing $10^{53}\mathrm{erg}$ of gravitational binding energy. Although CCSNe has been detected and studied across the electromagnetic (EM) spectrum, they cannot uncover the processes deep in the star’s core when the explosion ignites [9]. Therefore, studying CCSNe through the GW spectrum will unveil the processes contributing to this stellar explosion. In this article, we will focus on detecting GWs emerging from CCSNe.

The current understanding of CCSNe dynamics is based on two models: the neutrino-driven mechanism and the magneto-rotational mechanism. A multi-messenger study of CCSNe by combining GW and EM spectra will help settle and evaluate our understanding of the two current models used for simulating GWs from CCSNe. The two models are currently used in two-dimensional [10] and three-dimensional [11] cases to simulate the interaction between particles and generate the signatures of GWs. These simulations are computationally expensive and require multiple iterations to initiate a supernova explosion [12]. A portion of these simulations fail to achieve an explosion state, resulting in no CCSNe GWs generation [10]. In [13], 28.3% out of 1684 simulations failed to explode, and no signature was obtained. This is due to the many challenges facing the CCSNe simulation, where the resolution of the particles is crucial for a successful simulation. The resolution of particles will determine how challenging the other constraints of this simulation are, such as the interactions of the electron, muon, tau neutrinos, and their antiparticles with ordinary matter [14]. The resolution used to simulate these interactions also increases the complexity of the simulation, which needs to account for the relativistic effect, sophisticated equation of state (EOS), and more constraints to achieve realistic circumstances. Generating a bank of CCSNe waveforms is currently limited given the resources and computational power, which limits the number of GW templates to cover the parameter space of possible CCSNe events [13]. The limitation of template-based matching, i.e., the Coherent WaveBurst (cWB) pipeline proposed in [15], has prompted the exploration of alternative methods compared to template-based ones. Eventually, the new alternative methods may achieve the detection of the first CCSNe GW signal in the next decade.

Computer science has enabled the development and implementation of machine learning (ML) algorithms, i.e., algorithms that can learn from datasets and make predictions without being explicitly programmed. These algorithms have become popular nowadays in signal and noise processing [16]. ML techniques, particularly convolutional neural networks (CNNs), have been proposed and successfully tested on simulated data for the detection of CCSNe signals, being one of the novel ones, in the works of Astone et al. [14] and Chan et al. [17]. The recent literature has highlighted the potential of ML methods for gravitational wave detection. For example, Iess et al. [18] explored 1D and 2D CNNs, as well as LSTM architectures, for CCSNe detection using both time-series and spectrogram inputs on simulated interferometer noise. Their results confirm the utility of deep learning in noisy environments. However, they did not examine Q-transform spectrograms, nor did they address generalisation across unseen EOS models or detection at SNR levels below 1. Similarly, survey efforts, such as the one in Sasaoka et al. [19], which reviews deep learning for CCSNe detection, focus on summarising the breadth of ML approaches tested for this problem. While these works emphasise the diversity of ML tools, they remain primarily descriptive and do not establish quantitative comparisons between different time–frequency representations, such as STFT versus Q-transform. Additionally, in Marx et al. [20], the authors developed a machine learning pipeline for the real-time detection of compact binary coalescence. While this demonstrates the readiness of ML methods for integration into gravitational-wave astronomy pipelines, compact binary signals are well modelled, unlike the highly variable and weaker CCSNe signals. Thus, the unique challenges of CCSNe detection—waveform diversity, low SNR, and robustness to unseen EOS families—remain less explored.

The results mentioned above confirm that employing ML can significantly improve the detection sensitivity of gravitational wave signals by reducing false positives and filtering out noise events. In this article, we train two CNNs separately using time–frequency spectrograms from the short-time Fourier transform (STFT) and the Q-transform (QT) [21], computed from CCSNe GW signals and aLIGO noise data. While QT has been successfully utilised for parameter inference in gravitational wave data analysis, its application for detecting CCSNe signals represents a novel approach. We will compare the effectiveness of these two methodologies using datasets that include various EOSs and distances ranging from 0.1 to 10 kpc.

The main contributions of this paper can be summarized as follows: (i) we present the first direct comparison of short-time Fourier transform and Q-transform spectrograms for the CNN-based detection of CCSNe signals; (ii) we demonstrate the ability of our model to generalize across unseen EOS families, improving robustness to astrophysical variability; (iii) we show that our approach can reliably detect CCSN signals at extremely low signal-to-noise ratios (SNR ≤ 1), surpassing thresholds achieved in prior deep learning studies; (iv) we extend interpretability techniques (Grad-CAM) into the QT-based detection domain, particularly in low-SNR settings, to provide insight into what features the CNN uses for classification.

This paper is structured as follows. In Section 2, we review the work done on the detection of CCSNe and discuss recent alternatives with potential in this context. In Section 3, we describe how the simulated data is generated and our method for processing the data and detecting CCSNe. We present and discuss the results in Section 4. Finally, we conclude by summarising the results, discussing the limitations of the proposed methodologies, and outlining future work.

## 2. Literature Review

Since their appearance, ML techniques have shown their ability to enhance and bypass many difficulties in problem-solving, especially in signal processing. Many studies have demonstrated the possibility of training a CNN on time series and spectrograms to classify signals, e.g., [16]. GW astronomy has also incorporated machine learning techniques to enhance signal detection, especially for complex cases like searching for core-collapse supernovae signatures. The authors of [14] made the first steps in CCSNe detection by taking advantage of the peculiarities of these GW signals, particularly the monotonic rise in frequency related to g-mode excitation. In their study, the simulated g-mode signature was injected into Gaussian noise to imitate the spectral behaviour of LIGO. Then, the cWB pipeline was used to generate a time–frequency spectrogram, and a CNN was trained to classify these images of spectrograms into noise and noise + signal classes. This method offers a novel way to detect GWs from non-rotating or slowly rotating progenitor stars, expanding the scope of detectable GW events. On the other hand, ref. [17] explored the use of CNNs for the classification of GW signals from CCSNe. The CNN was tailored for multi-class classification, distinguishing between background noise and signals from different types of supernovae explosions, whehter magneto-rotational or neutrino-driven, embedded in background noise. In addition to aLIGO data, the CNN was trained with AdVirgo and KARGA, using a categorical cross-entropy loss function. The combination of four detectors allowed the authors to evaluate detections of potential extragalactic CCSNe GW events at 200 kpc. The authors of [22] made a significant step forward in using supervised ML for GW detection from CCSNe. The cWB pipeline was integrated with ML classifiers like linear discriminant analysis (LDA) and support vector machines (SVMs). The classifiers were trained on features of the reconstructed GW burst, such as duration, central frequency, and detection statistics provided by the cWB pipeline. The classification was done independently on each of the LIGO detectors. The CCSNe models considered distances ranging from 1 to 10 kpc. Recently, ref. [23] tested deep learning techniques in the time domain and neural networks in the time–frequency domain to classify and infer on the CCSNe parameters. The study achieved better detection levels in the time domain, reaching 98% for a signal-to-noise ratio (SNR) greater than 10. These studies have demonstrated the potential of machine learning techniques in unravelling the complex nature of CCSNe and enhancing the sensitivity and accuracy of GW detection. Another recent survey of deep learning approaches for CCSNe GW detection is the one in Sasaoka et al. [19], which emphasises the range of methods tested, but the survey tends to remain descriptive rather than pushing new detection thresholds in noisy regimes. In a broader context, the MLy [24] pipeline leverages CNNs to detect modelled gravitational-wave bursts, including signals from CCSNe, within the LIGO-Virgo network. By analysing both strain data and the Pearson cross-correlation between detectors, MLy achieves detection efficiencies comparable to traditional methods while significantly reducing computational costs and enabling real-time alerts for multi-messenger astronomy.

The difficulty in detecting CCSNe signals lies, in part, in the need for robust signal extraction methods. Recent studies, such as [25,26], have made significant contributions to the development of adaptive neural control systems for noise reduction and signal tracking, laying the groundwork for foundational methods that can be extended to GW analysis. Similarly, ref. [27] proposed a reinforcement learning-based strategy, namely Q-Learning, for iterative optimisation in complex system identification, offering a pathway for improving the detection pipeline for faint astrophysical signals. Recent advances in neural-network-based classification techniques (e.g., [28,29]) present diverse opportunities for improving CCSNe detection, such as novel strategies for effective feature selection, pooling strategies to avoid over-fitting, and the use of Fourier CNNs for pattern detection in real-time applications, among others. The literature on algorithms with potential for CCSNe detection is extensive but remains largely unexplored.

## 3. Methods

First, we describe the CCSNe aLIGO [4] data generation process in the time domain used in this study. Then, we discuss the pre-processing of data before generating spectrogram images using the popular STFT, one of the most frequently used tools in time–frequency analysis, and the QT, a very popular technique in aLIGO GW time–frequency data analysis. Finally, we describe the CNN proposed for CCSNe detection, including its architecture and the way it is trained, validated, and tested.

### 3.1. Data Generation

**Figure 2:**
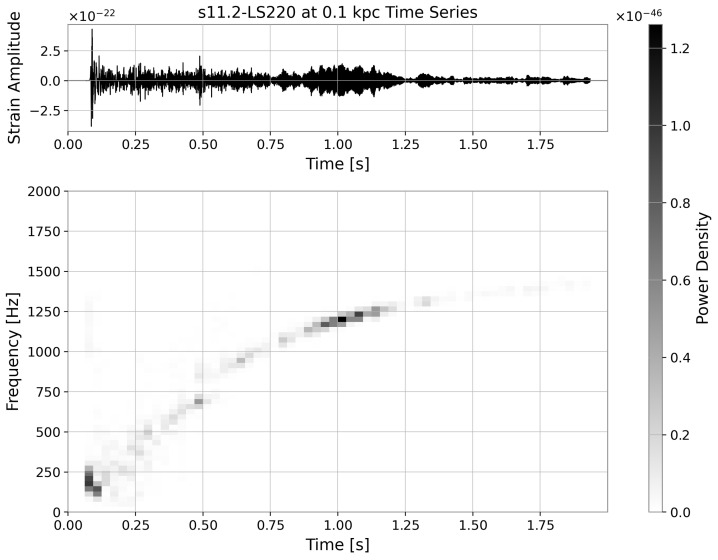
(**Top Panel**): CCSNe’s GW signature at 0.1 kpc embedded in the aLIGO noise. (**Bottom Panel**): The STFT spectrogram of the GW signal.

The positive class contains simulated CCSNe GW signals from eight EOS models: *s11.2, s15, s20, s25, and s40* under *LS220*, *s15* under *GShen*, and *s15 and s20* under *SFHo*, where the number following the “s” denotes progenitors with zero-age main sequence mass [10]. The simulated waveforms are submerged by the aLIGO noise [30], with all being two seconds long, pre-whitened, and sampled at $2^{14}$ Hz. We apply zero padding to the simulated waveforms that are under two seconds long. The original waveform is attenuated inversely proportional to the source distance. We consider twenty-one distances from 0.1 to 10 kpc with 0.5 kpc increments from 0.5 kpc, i.e., {0.1,0.5,1.0,…,10.0}. A single realisation in the time domain is presented in Figure 2, accompanied by its time–frequency representation obtained using the STFT.

**Figure 3:**
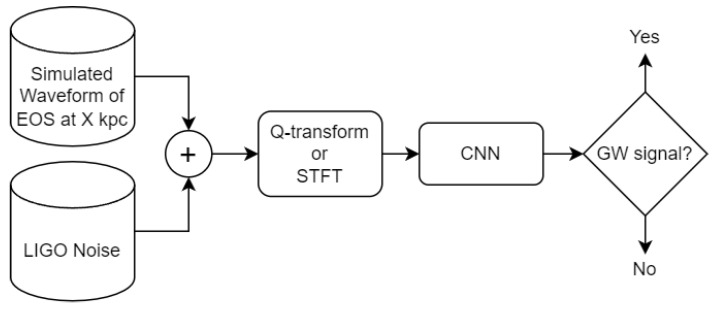
Data generation and analysis process. The noise data is generated by omitting the inclusion of the simulated waveform.

The signal is submerged in a random aLIGO noise realisation generated from its power spectral density. This process is repeated 100 times for each waveform and distance. Part of the procedure is illustrated in Figure 3. The time–frequency transformation (either STFT or Q-transform) produces a magnitude response in the form of an image to the CNN input. This procedure results in a total of 100 realisations × 8 models × 21 distances = 16,800 CCSNe signal observations. These observations are divided later into training, validation, and testing datasets. The split is as follows: 5 models for training, 1 for validation, and 2 for testing. See Table 1 for more details.

The negative class, i.e., aLIGO noise without any GW signals, is generated using the aLIGO power spectral density [4]. The same number of 16,800 noise realizations was generated to balance positive and negative classes. In this work, we use the standard matched filter signal-to-noise ratio [31] (SNR) to characterize each injected waveform. For any two strain time series a(t) and b(t), their noise weighted inner product is defined as

(1) (a|b)=4Re∫0∞a˜(f)b˜*(f)Sn(f)df,

where a tilde denotes the Fourier transform, an asterisk denotes complex conjugation, and $S_{n}\left(f\right)$ is the one-sided power spectral density of the aLIGO design noise. For a template waveform h(t), the optimal SNR is

(2) $$\rho_{\mathrm{opt}}=\sqrt{\left(h|h\right)}.$$

When the signal h(t) is added to Gaussian noise n(t) drawn from the same $S_{n}\left(f\right)$ so that the data are s(t)=h(t)+n(t), the matched filter SNR in the data is

(3) $$\rho =\frac{\left(s|h\right)}{\sqrt{\left(h|h\right)}}.$$

In our injections, the waveforms differ only by the source distance *d*, so the SNR scales as

(4) $$\rho \propto \frac{1}{d}.$$

**Figure 4:**
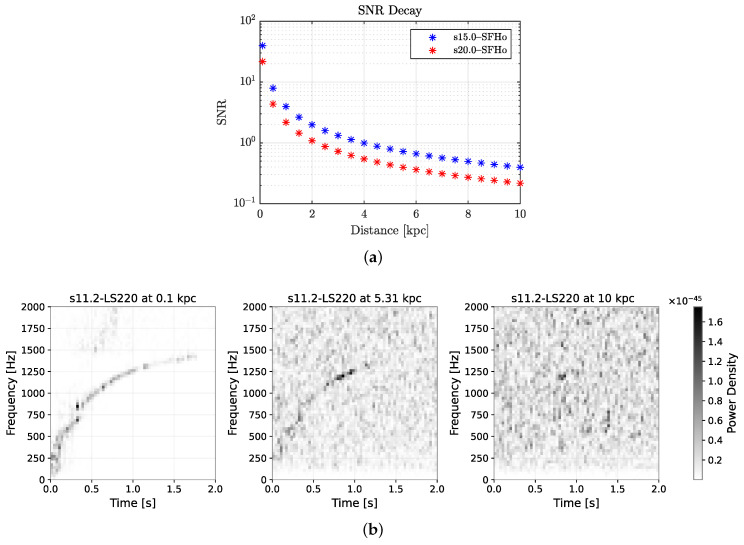
Signal decay over distance. (**a**) SNR of two signals across distances (0.1–10 kpc). (**b**) STFT spectrogram of s11.2-LS220 observed at 0.1, 5.31, and 10 kpc.

The SNR across distances varies for the 8 EOS models, resulting in different degrees of difficulty for detection. To illustrate this, the SNR of two GW signals against the source distance is plotted in Figure 4a. The initial SNR for *s15–SFHo* and *s20–SFHo* is 39.6 and 21.7 at 0.1 kpc, and the SNR at 10 kpc is 0.396 and 0.217, respectively. Their SNR profiles entail a more challenging signal detection task because when compared to a similar study in [14], their smallest SNR is 8, while our SNR drops below 1 when the source distance is beyond 4 and 2 kpc for *s15–SFHo* and *s20–SFHo*, respectively. Signals from these two EOS models are later used as the test set in the performance measurement of the trained deep convolutional neural network for CCSNe signal detection. Figure 4b presents the STFT spectrograms for CCSN signals from the *LS220* EOS family, which exhibit higher SNR retention at larger distances. At 5 kpc, the time–frequency ridge appears sparse yet remains discernible to the human eye, whereas at 10 kpc, it becomes largely indistinguishable from the noise background. Even under optimistic conditions—Gaussian noise, perfect whitening, and the high-SNR EOS family—the recovery of CCSN signals remains challenging at greater distances.

In this study, we adopt a single-detector framework to enable robust CCSN signal detection at increased distances. Although incorporating additional detectors and combining their outputs would likely enhance sensitivity and reduce the false-detection rate, isolating a single detector allows for a clearer assessment of the intrinsic feasibility of detection. This approach thus establishes a rigorous baseline for evaluating the difficulty of detection.

### 3.2. Data Processing

In this study, we use STFT and QT to produce spectrograms. STFT is widely used in time–frequency analyses. We refer the reader to the extensive literature for its details, and instead, we focus on describing the QT. QT has become an essential component in the aLIGO pipeline for searching gravitational wave bursts [21]. Initially, it was applied in the field of music signal processing to differentiate similar notes played simultaneously [32]. The QT is a time–frequency transform designed to represent how the frequencies of a signal vary over time. This transform employs Gaussian windowed sinusoids to analyse signals, balancing time and frequency resolutions. Our study uses a discrete version of the QT [21], incorporating a Hann window in the frequency domain. The “Q” in Q-transform refers to the Q factor, which measures the window’s width relative to its centre frequency, enabling fine-tuning of the resolution. A high Q-factor indicates a narrow window in the time domain and a broader window in the frequency domain.

The time-series signal from aLIGO, denoted as x(t), is projected onto the w(t−τ,f) windowed complex sinusoids of frequency *f* centred around time τ, expressed mathematically [21] as

(5) $$x\left(\tau ,f\right)=\int_{-\infty }^{+\infty }x\left(t\right)w\left(t-\tau ,f\right)e^{-i2\pi ft}dt.$$

For computational efficiency, the QT can alternatively be represented using the Fourier transform of the data time series as

(6) $$x\left(\tau ,f\right)=\int_{-\infty }^{+\infty }\tilde{x}\left(\phi +f\right)\tilde{w}\left(\phi ,f\right)e^{+i2\pi \phi \tau }d\phi ,$$

where ϕ is the frequency shift [33]. This formulation allows the Fourier transform (FT) to be computed once. Subsequently, the Q-transform is calculated using this precomputed FT, applying a frequency shift and the window function in the frequency domain before inverting the FT. The QT used in our analysis is specifically adapted for aLIGO noise, normalising the window to counteract the power spectral density of detector noise and accurately recovering the energy of transient bursts. This Q-transform formulation is a discrete implementation of the continuous wavelet transform (CWT) utilizing complex Morlet wavelets [21]. By maintaining a constant quality factor *Q*, the transform inherently performs a multi-resolution analysis similar to standard wavelet approaches, providing optimal time resolution for high-frequency transients and frequency resolution for low-frequency structures.

**Figure 5:**
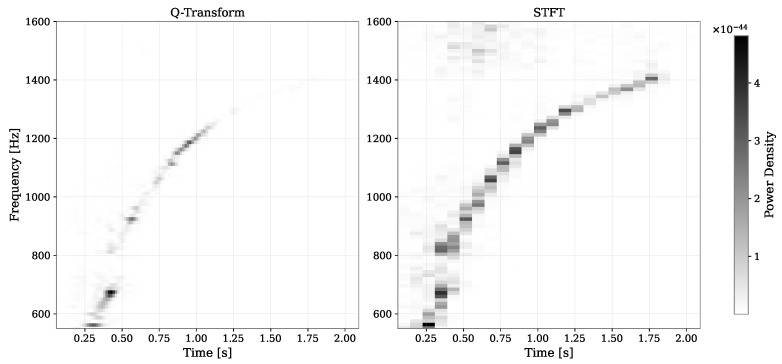
Comparison of QT and STFT spectrogram outputs of a simulated CCSNe event for the same frequency range. The STFT spectrogram has been truncated for comparison purposes. Notice how the QT output has a higher resolution in both time and frequency axes.

In this study, we utilise the QT implementation from the GWpy Python package [31] with specified parameters: *qrange* 100 to 200, *frange* 0 to 1600 Hz, *tres* 2/128 s, *fres* 1600/128 Hz, and *whiten* = False. The term *qrange* refers to the range of the Q-factor used in the QT, and it is related to the time and frequency spread of the signal. A high Q-factor corresponds to a signal that is narrow in frequency but spread out in time, whereas a low Q-factor corresponds to a signal that is narrow in time but spread out in frequency. *Frange* is the range of frequency used to compute the QT. *tres* and *fres* are the time and frequency resolutions of the output spectrogram from QT, respectively, and they determine the size of the spectrogram. The remaining parameters are left at their default settings. The *frange* was selected based on the observation that all eight equations of state models studied exhibit frequency characteristics within this range. Prior to applying the QT, we band-pass the signal between 100 and 2000 Hz to attenuate irrelevant frequency components. This procedure is similarly applied to the noise input. It is important to note that this QT method interpolates the output to produce a high-resolution spectrogram on both time and frequency axes, allowing independent adjustments of *tres* and *fres*. Figure 5 demonstrates the superior time and frequency resolution of the QT compared to the spectrogram output from the short-time Fourier transform, particularly for the *s11.2–LS220* model at 0.1 kpc. The high-resolution images from the QT are directly used as inputs for the convolutional neural network, which is discussed in the following section.

### 3.3. Deep Convolutional Neural Network

Since our focus is on detecting the CCSNe GW events from the aLIGO interferometer using the STFT and QT outputs, in 2 independent analyses, this is turned into an image classification problem. A similar work in [34] also used time–frequency spectrograms as inputs to classify human activity using CNN. We use transfer learning based on ResNet-18 [35] as the base network, leveraging its pre-trained parameters to alleviate training challenges and harnessing its capability to capture relevant lower-level features across domains. The choice of ResNet-18 in this study is intentional despite the existence of more recent architectures. ResNet introduced the concept of residual learning and skip connections, which stabilise convergence and mitigate vanishing gradient flows during backpropagation [35]. Its moderate depth provides a good balance between model capacity and computational efficiency, making it suitable for training on relatively small and noise-dominated datasets such as LIGO signals. The use of batch normalisation [36] and ReLU activations [37,38] further enhances training stability and nonlinearity, while pretraining on large image datasets allows effective transfer learning with limited astrophysical data. Overall, ResNet-18 offers a reliable and interpretable baseline for benchmarking future, more complex models in gravitational-wave detection.

**Figure 6:**
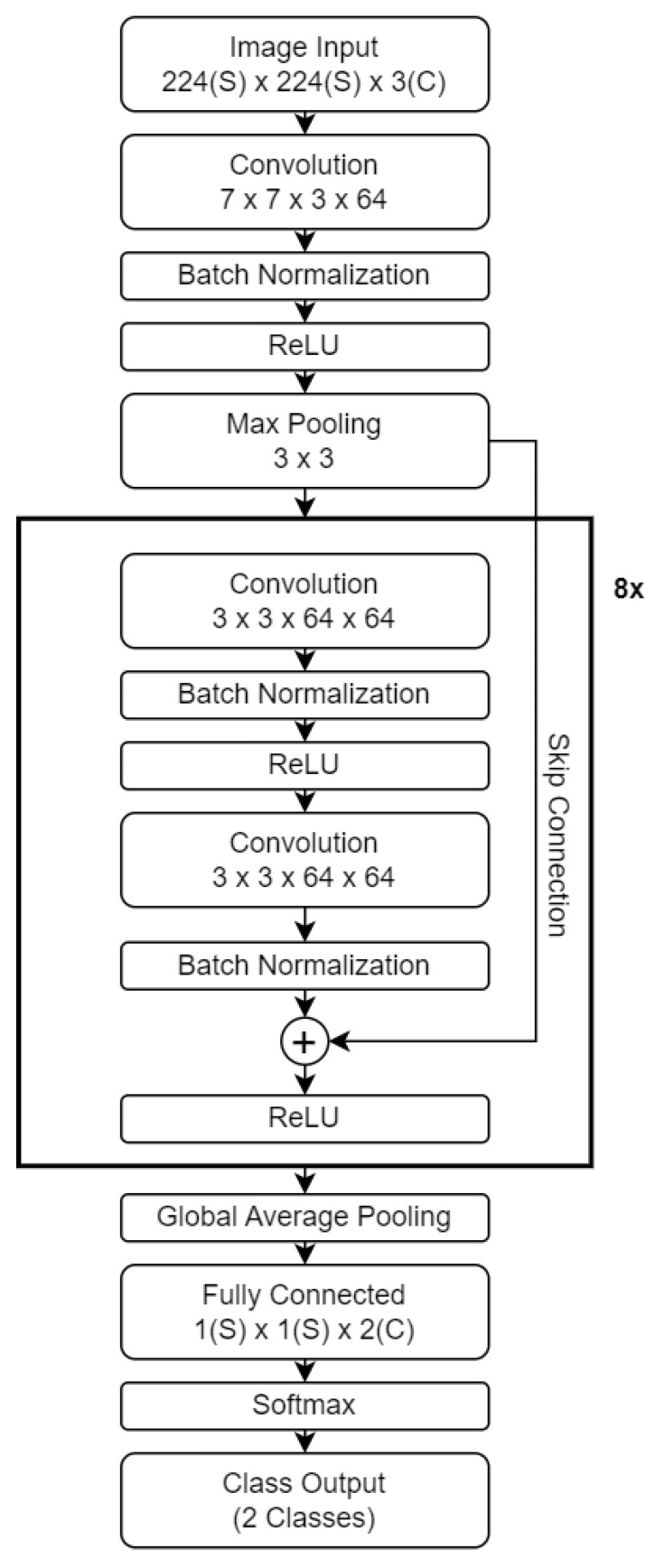
The architecture of the adapted deep convolutional neural network ResNet-18. The last fully connected layer is modified to suit the number of classes.

Our network architecture, illustrated in Figure 6, processes input spectrograms resized to 224×224×3 (width × height × channels). The configuration follows the original ResNet–18 design, incorporating a 2D convolutional layer with a 7 × 7 kernel, batch normalisation, and ReLU activation, as mentioned before. However, the final fully connected layer is modified to have 2 neurons, corresponding to the two classes of interest: event signal and the aLIGO noise. We keep the *softmax* activation function to output class probabilities for the positive and negative classes $P_{s}$ and $P_{n}=1-P_{s}$, respectively, that sum up to one. In the last class output layer, the prediction is made based on the positive class threshold *T*, which is between 0 and 1. If $P_{s}\geq T$, then the class belongs to a signal; otherwise, it belongs to the noise.

The dataset is divided into training, validation, and testing splits, as summarised in Table 1. Each entry in the table has 2100 signal instances. The training and test split was performed to separate the EOSs; thus, only *LS220* was used for training, *GShen* was used for validation, and *SFHo* was used for testing. This ensures that the CNN trained with one EOS is able to carry out detections with other EOSs. The same number of negative class instances was generated, so the trained network is not biased towards either class. To simulate the timing uncertainty inherent in actual detection scenarios and bolster the CNN’s robustness against overfitting, we employ random horizontal image translations up to 50% of the image width as part of our data augmentation and neural network regularisation strategy. The spectrogram *h* is rescaled to the range between 0 and 255 via

(7) $$h_{\mathrm{rescale}}=255\times \frac{h-\min(h)}{\max(h)-\min(h)}.$$

The rescaled spectrogram is then treated as a single-channel grey-scale image input to the CNN.

**Figure 7:**
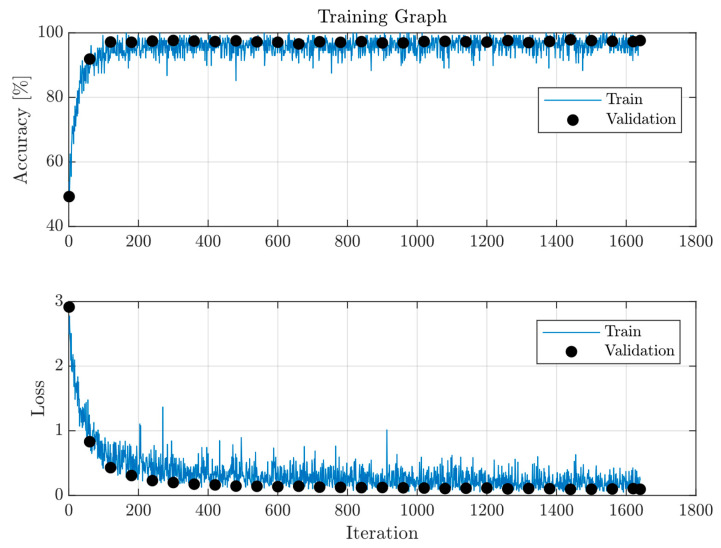
Accuracy and loss plot of the training process of QT-CNN, with validation data *s15–GShen*.

For training, we employ the Adam optimiser [39] with a learning rate of $10^{-6}$, applied over 10 epochs, and include $L_{2}$ regularisation set at 0.05. The network, which trains mini batches of 128 training samples, does not freeze any layers, allowing all layers to update during training. These hyperparameters were tuned such that small and similar training and validation losses were obtained. This setup achieved a final validation accuracy of 97.95%, which is very close to the final training accuracy. This indicates a successful optimisation and learning generalisation. Training progress, represented in Figure 7, plots classification accuracy and network loss over training iterations. The training converges in 400 iterations. We utilise weighted binary cross-entropy loss to penalise false negatives more heavily, enhancing the network’s sensitivity to genuine GW signals. This is given by

(8) $$\mathrm{Loss}=-\frac{1}{N}\sum_{i=1}^{N}\left(w_{p}\cdot y_{i}\cdot \log\left(P_{s,i}\right)+w_{n}\cdot \left(1-y_{i}\right)\cdot \log\left(1-P_{s,i}\right)\right),$$

where $w_{p}=5$ and $w_{n}=1$ represent the weights for positive and negative class errors, respectively, with *N* denoting the mini-batch size at 128 and $y_{i}$ denoting the binary indicator of whether the signal instance is positive (1 for CCSNe signal) or not (0 for aLIGO noise). The ratio of weights $w_{p}$ to $w_{n}$ indicates that we are penalising false negative classifications more, where a true CCSNe signal is misclassified as aLIGO noise.

**Figure 8:**
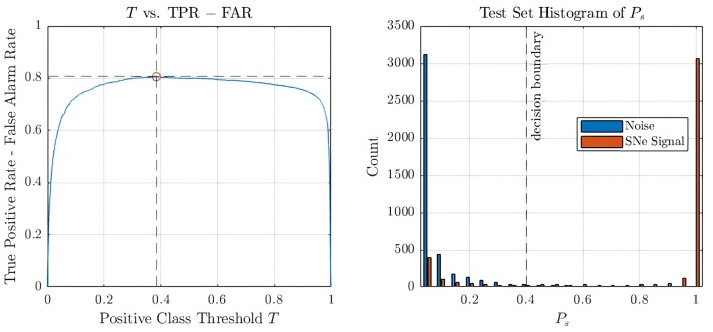
(**Left**): Line plot of the difference between TPR and FAR (solid blue line) as a function of the positive-class threshold *T* for the QT–CNN case. The red marker at T≈0.4 indicates the threshold at which this difference is maximized. (**Right**): Histogram of $P_{s}$ across all test-set images. The dashed vertical line corresponds to the threshold obtained from the left panel.

For the CNN trained using the spectrograms produced by QT, which we call QT-CNN, the positive class threshold *T* is computed by maximising the difference between true positive rate (TPR) and false alarm rate (FAR), which means maximising TPR and minimising FAR. The relationship is plotted on the left of Figure 8, and it indicates that when $P_{s}\geq 0.4$, the input is classified as a CCSNe signal. Therefore, for the QT-CNN, the threshold is T=0.4. The classification threshold for the CNN trained using spectrograms produced by STFT, which we call STFT-CNN, is set by default at T=0.5.

After hyperparameter tuning, the network was trained again with training and validation data combined. The classification results from the test set are presented and discussed in the following section.

## 4. Results and Discussion

**Figure 9:**
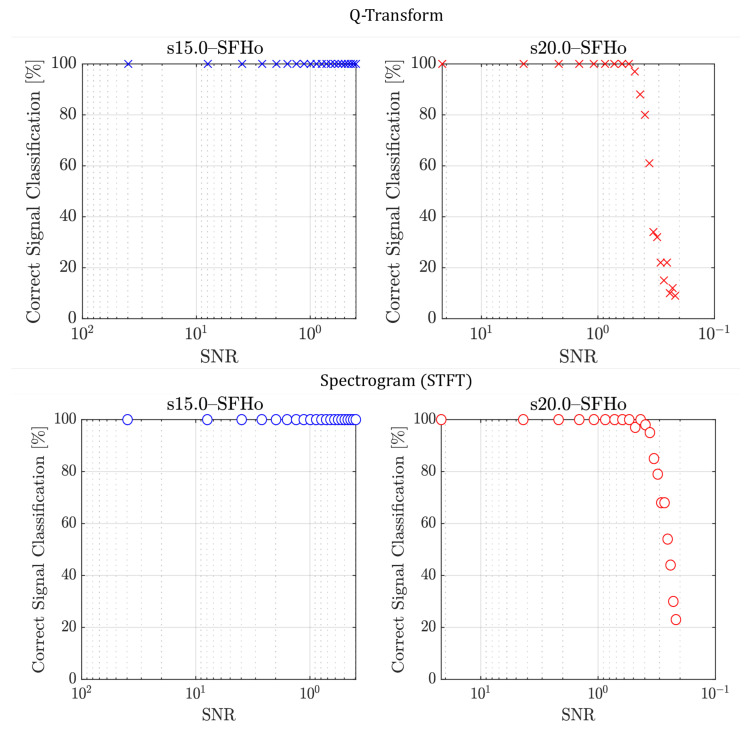
(**Top**): True positive rate for *s15.0–SFHo* and *s20.0–SFHo* against SNR using the QT as the input to the CNN. There are 2100 test images for each EOS model; positive class threshold T=0.4. (**Bottom**): True positive rate using the STFT spectrogram as the input; positive class threshold T=0.5.

The output spectrograms from QT and STFT have been used as the inputs to train the CNNs. We denote the CNNs as QT-CNN and STFT-CNN, respectively. The training procedures are the same for both models. TPR is first tested to get a quick overview of the model’s ability to classify the signal correctly. It is plotted in Figure 9 for the two signals in the test set: *s15.0–SFHo* and *s20.0–SFHo*. It is expressed as the percentage of correct signal classifications over the total number of signal instances at a particular distance. In the QT-CNN case, for *s15.0–SFHo*, all signals are correctly predicted up to 10 kpc, where the SNR is just below 0.5. A steady, high true positive rate is observed for *s20.0–SFHo* at an SNR greater than 0.5, where the distance is 4 kpc. Beyond this SNR, the performance starts to drop as expected, which is not surprising considering the SNR at 10 kpc is just above 0.2. It can be observed that regardless of the EOS, the network is capable of correctly identifying the event signal at a high success rate for an SNR as low as 0.5. When comparing our result to that of [14], we achieved a TPR of 100% for an SNR as low as 0.5 from the *s15.0–SFHo* model where they also achieved the same TPR but at an SNR of 20 (interpolated from Figure 8 (left)). In the STFT-CNN case, the CNN performs equally well for the *s15.0–SFHo* model but outperforms QT-CNN slightly for the *s20.0–SFHo* model at low SNRs, and it achieves a TPR just above 20% for an SNR of approximately 0.2.

**Figure 10:**
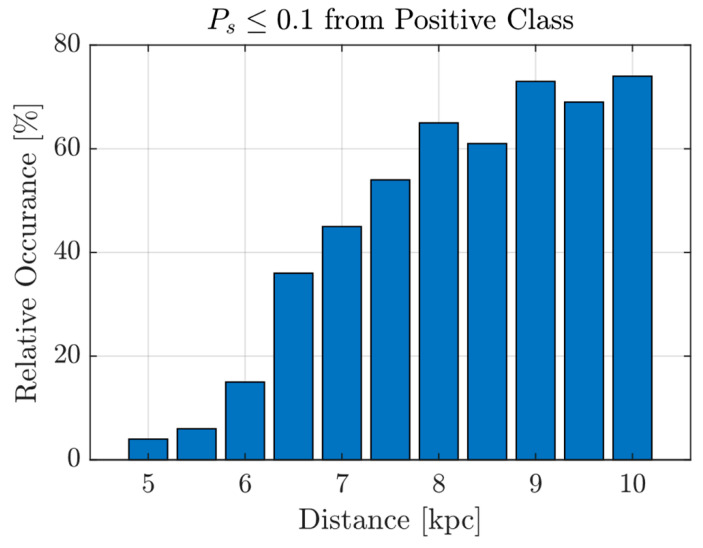
Relative occurrence of test samples from QT-CNN, for which its $P_{s}\leq 0.1$ is from the *s20.0–SFHo* model.

To further analyse the network’s confidence in classifying these two classes, a histogram of all positive class probabilities $P_{s}$ is shown on the right of Figure 8. The histogram indicates a clear separation of the classes, with most instances of each class distributed at opposite ends of the histogram. This observation is in agreement with the insensitivity of the change in *T* to TPR–FAR around the middle plateau, suggesting a stable binary classification model. However, it is worth noting that there are a number of false negatives at $P_{s}\leq 0.1$, consisting mostly of signals from beyond 6 kpc in the *s20.0–SFHo* model. A bar graph of their relative occurrence is plotted in Figure 10, confirming the expected behaviour: as the distance increases, the SNR decreases approximately inversely with distance, causing the detection rate to drop.

The overall test set performance for all source distances is summarised in the confusion matrix presented in Table 2. The values are expressed as percentages of the ground truth. We used the following metrics to evaluate the performance of the CCSNe GW signal classifier:

(9) $$\begin{array}{cc} \; & \mathrm{True}\;\mathrm{Positive}\;\mathrm{Rate}\;\left(\mathrm{TPR}\right)=\frac{\mathrm{TP}}{\mathrm{TP}+\mathrm{FN}}, \end{array}$$

(10) $$\begin{array}{cc} \; & \mathrm{False}\;\mathrm{Alarm}\;\mathrm{Rate}\;\left(\mathrm{FAR}\right)=\frac{\mathrm{FP}}{\mathrm{FP}+\mathrm{TN}}, \end{array}$$

(11) $$\begin{array}{cc} \; & \mathrm{Precision}=\frac{\mathrm{TP}}{\mathrm{TP}+\mathrm{FP}}, \end{array}$$

(12) $$\begin{array}{cc} \; & F_{1}\;\mathrm{score}=2\times \frac{\mathrm{precision}\times \mathrm{TPR}}{\mathrm{precision}+\mathrm{TPR}}. \end{array}$$

For more information about these metrics, see, for example, [40]. A TPR of 82.9% and 91.5% was achieved for QT-CNN and STFT-CNN, respectively, over an SNR range from 39.6 to 0.2. For the QT-CNN, out of 4200 aLIGO noise and true event signal instances, 4092 were correctly rejected as noise, and 3482 were correctly identified as true event signals. This is equivalent to an overall true negative rate of 97.4%, a TPR of 82.90%, a FAR of 2.6%, a precision of 0.970, and an $F_{1}$ score of 0.893. The TPR of 82.90% is higher than the 69% obtained in [23], despite our much lower SNR of 0.1 at 10 kpc compared to their SNR of 20.

For the STFT-CNN, a TPR of 91.5% outperforms that of the QT-CNN, mainly due to the difficult instances of signals at a very low SNR. It also results in a higher precision of 0.977 and a higher $F_{1}$ score of 0.945. This might reflect how using a single Q-range limits the frequency bandwidth of the input data and how a fixed Q-range might not be optimal for all EOSs. On the one hand, the STFT spectrogram data contain the full frequency bandwidth of the CCSNe signal. On the other hand, the QT-CNN is penalised when using a single Q-range for all the EOSs, which limits the frequency bandwidth and excludes a significant portion of the low and high frequencies of the CCSNe signal. Given the current experimental setup, STFT-CNN outperforms QT-CNN, detecting 8.6% more event signals, using the default class threshold of T=0.5. Most of the 8.6% signals lie below SNR =0.5, as shown in Figure 9.

**Figure 11:**
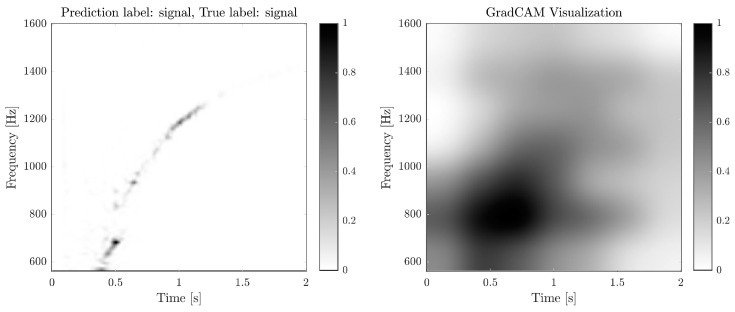
(**Left**): QT spectrogram for a signal observation. (**Right**): Grad-CAM visualisation for the signal class prediction from the QT-CNN. The network primarily focuses on the region where the signal signature is most pronounced.

**Figure 12:**
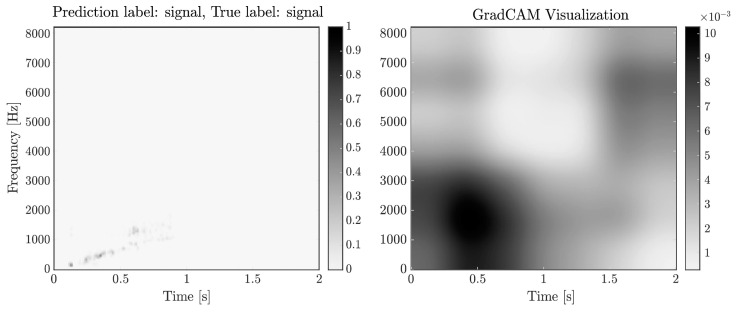
(**Left**): STFT spectrogram for a signal observation. (**Right**): Grad-CAM visualisation for the signal class prediction from the STFT-CNN. The network concentrates most around the signal signature at 0.5 s and 1500 Hz.

To better understand the network’s decision process and ensure that the classification made is justifiable, we examine the network with Grad-CAM visualisation [41] for one realisation from the predictions of both networks, shown in Figure 11 and Figure 12. This method gives an idea of which parts of the input image are most relevant to a prediction. It reveals that the QT-CNN focuses correctly on a blob centred around 0.6 s and 800 Hz, where the signal’s time–frequency signature is most likely to appear. Similar results are obtained for the STFT-CNN, where the lower left region of the input contributes the most to the prediction of the signal class. However, note that the area above 1600 Hz is relevant for the prediction, a region that was omitted in the QT-CNN and could explain the difference in their performance.

## 5. Conclusions

Detecting GWs from CCSNe could be the next breakthrough for ground-based detectors, underscoring the critical importance of advancing detection techniques. In this paper, we have shown that a CNN can effectively detect these GW signals in the time–frequency domain. We investigated two pre-processing methods for the time–frequency domain analysis to train the CNNs: STFT and the QT. Both approaches, QT-CNN and STFT-CNN, perform similarly in terms of noise and signal identification down to an SNR of 0.5 in our test set. However, STFT-CNN outperforms QT-CNN in detecting signals below an SNR of 0.5. The TPRs are 0.83 and 0.92 for QT-CNN and STFT-CNN with respect to an SNR as low as 0.2, respectively. STFT-CNN also achieved a higher precision of 0.977 and a higher $F_{1}$ score of 0.945.

Although STFT-CNN yielded better results for faint signals (those at extreme low SNRs), the experimental setup was designed for a singular CNN architecture to test both methods. This setup resulted in using one fixed Q-range to produce one QT spectrogram for each different data, which focuses on signal features in one portion of the whole frequency band. The choice of multiple Q-ranges to process each data will potentially help cover the entire CCSNe signal features and the whole frequency band. Therefore, a CNN architecture adapted for multiple QT spectrograms for each signal could potentially improve the CNNs performance in detecting GWs from CCSNe. Alternatively, employing higher-complexity wavelet bases instead of the QT could enhance the feature resolution of the training spectrograms and potentially allow for the capture of more subtle signal morphologies.

Our experiment was conducted using only simulated CCSNe gravitational-wave signals and aLIGO noise. Consequently, the performance of our CNNs in the presence of real detector noise and transient artifacts (glitches) remains to be evaluated. This represents a natural limitation of this study and a promising avenue for future research. In particular, glitches—short-duration, non-astrophysical noise transients—are common in real interferometer data and can mimic true astrophysical signals, especially faint CCSNe signals, posing additional challenges for classification. Although we acknowledge that real detector noise and glitches may influence detection performance, they also provide an opportunity to test the robustness and adaptability of our models under more realistic conditions. To partially address this, we employed the aLIGO power spectral density model [30], which captures key statistical features of the instrument noise. As part of our future work, we plan to extend the present framework to include real aLIGO data and systematically incorporate glitch classes into the training and testing processes to better emulate operational conditions of current and next-generation detectors.

Similarly, in this study, we focused on data from a single interferometer (LIGO Livingston) to allow a controlled and detailed evaluation of the proposed detection framework. Although multi-detector analyses—such as combining data from LIGO Hanford, Virgo, or KAGRA—can further enhance sensitivity and improve source localisation, our goal here is to demonstrate the feasibility of detecting core-collapse supernova signals from a single detector’s output. This scenario is not only representative of situations where detectors may be offline or operating asynchronously but also provides a clear baseline for assessing model performance. The proposed framework can be readily extended to multi-detector configurations through coincidence detection or network-level feature fusion, which is also part of our future work.

Our CNNs, like other similar algorithms, lack interpretability. To address this, we used Grad-CAM visualization to highlight the relevance of spectrogram regions in the prediction process, although the exact decision-making remains unclear. Nevertheless, CNNs are well-suited when prediction is the primary focus of the analysis. In particular, they are especially useful for CCSNe detection, as they do not require a theoretical background for training.

We have demonstrated that CNNs trained in the time–frequency domain are viable methods for CCSNe detection at low SNRs. Our findings highlight the potential for exploring new techniques. Recent advances in neural-network-based classification techniques (e.g., [25,26,28,29]) present diverse opportunities for improving CCSNe detection. Similarly, the development of 3D simulations for CCSNe GW signals will provide more datasets and enable the training of CNNs in more realistic scenarios, improving detection accuracy. Additionally, a fast emulator for CCSNe signals [42], based on a deep convolutional generative adversarial network, has recently been proposed and may prove helpful in developing advanced detection methods. The incorporation of these new developments will play an integral role in future work.

## Figures and Tables

**Table 1:** Train, validation, and test split of the data. The same number of 2100 instances of aLIGO noise was also included for each EOS model.

EOS	LS220					GShen	SFHo	
Model	s11.2	s15	s20	s25	s40	s15	s15	s20
Train	2100	2100	2100	2100	2100	-	-	-
Validation	-	-	-	-	-	2100	-	-
Test	-	-	-	-	-	-	2100	2100

**Table 2:** Confusion matrix of the test set; positive class threshold of T=0.4 for QT-CNN and T=0.5 for STFT-CNN. The percentages show the proportion of positive/negative detections relative to the ground truth. The lowest SNR in the test set is 0.2 from s20.0 to SFHo at 10 kpc, as can be seen in Figure 4.

	Prediction				
		QT-CNN		STFT-CNN	
		aLIGO Noise	Event Signal	aLIGO Noise	Event Signal
Truth	aLIGO Noise	97.4%	2.6%	97.8%	2.2%
	Event Signal	17.1%	82.9%	8.5%	91.5%

## Data Availability

The original contributions presented in this study are included in the article. Further inquiries can be directed to the corresponding author.

## Abbreviations

aLIGO	Advanced Laser Interferometer Gravitational-Wave Observatory
CCSNe	Core-collapse supernovae
CNN	Convolutional neural network
cWB	Coherent WaveBurst
EM	Electromagnetic.
EOS	Equation of state
FAR	False alarm rate
GW	Gravitational wave
ML	Machine learning
QT	Q-transform
SNR	Signal-to-noise ratio
STFT	Short-time Fourier transform
TPR	True positive rate
